# High Accuracy Stool Biomarkers of Precancerous Colorectal Cancer Identified Using a 2000-Plex Immunoproteomic Screen

**DOI:** 10.1016/j.mcpro.2025.101079

**Published:** 2025-09-29

**Authors:** Kamala Vanarsa, Jessica Castillo, Hao Li, Kala T. Pham, Maria Akter, Sravya Gude, Kyung Hyun Lee, Claudia Pedroza, Robert Bresalier, Nicholas Chia, Chandra Mohan

**Affiliations:** 1 Department Biomedical Engineering, University of Houston, Houston, Texas, USA; 2 Department of General Surgery, Shanghai Tenth People's Hospital, Tongji University School of Medicine, Shanghai, China; 3 Department of Biology and Biochemistry, University of Houston, Houston, Texas, USA; 4 Center for Clinical Research and Evidence-based Medicine, McGovern Medical School, UT Health Science Center at Houston, Houston, Texas, USA; 5 Department of Gastroenterology, Hepatology and Nutrition, The University of Texas MD Anderson Cancer Center, Houston, Texas, USA; 6 Department of Surgical Research, Mayo Clinic, Rochester, Minnesota, USA

**Keywords:** colorectal cancer, stool, biomarker, proteomics, adenoma

## Abstract

Given the morbidity and mortality associated with colorectal cancer (CRC), novel biomarkers are clearly warranted, especially for early detection. An antibody-based screen of 2000 proteins was utilized to identify stool proteins that were elevated in patients with CRC, compared with healthy controls (HCs). Thirty-seven lead candidates were selected for ELISA validation in three independent cohorts comprised of CRC patients, advanced adenoma patients, and HCs, drawn from two different ethnicities. Of the 2000 proteins interrogated, 116 were differentially expressed in CRC stool, with 45 being elevated at twofold or higher; 37 of these proteins were selected for ELISA validation in three independent patient cohorts. Stool matrix metalloproteinase (MMP)-8, MMP-9, hemoglobin, Peptidoglycan Recognition Protein-S (PGRP-S), haptoglobin, and fibrinogen emerged as being most discriminatory for distinguishing CRC from HCs (area under the curve, 0.91–0.95), across cohorts and ethnicities, with several of these being significantly higher in more advanced stages of CRC. Stool fibrinogen, MMP-9, hemoglobin, MMP-8, and PGRP-S were the top 5 stool proteins with the highest accuracy for distinguishing advanced adenoma from HC, with stool fibrinogen topping the list with a receiver operating characteristic area under the curve value of 0.86. Functional pathway analysis revealed a significant over-representation of pathways related to antioxidant activity, integrin/receptor binding, cytokines, blood coagulation, and lipoprotein biosynthesis in patients with CRC compared with HC. Nuclear factor IC and IKZF2 were identified as key regulators of the molecular cascades over-represented in CRC. Stool fibrinogen, MMP-8, MMP-9, PGRP-S, haptoglobin, and myeloperoxidase emerge as promising biomarkers for distinguishing CRC/advanced adenomas/healthy stools, meeting or outperforming current yardsticks.

## Highlights

• First unbiased immunoproteomic screen of 2000 stool proteins in colorectal cancer (CRC), representing the largest such proteomic screen in this disease.
• Thirty-seven lead candidates were selected for ELISA validation in three independent cohorts, representing the largest ELISA validation program involving CRC biosamples.
• Report several stool biomarkers that detect advanced adenomas with significantly improved accuracy, with stool fibrinogen topping the list with a diagnostic accuracy of 86%, easily eclipsing the diagnostic performance of current tests.
• Uncovered several stool proteins with improved diagnostic accuracy for CRC as well as stool proteins that discriminate CRC from advanced adenomas.

## Graphical Abstract

**Figure:**
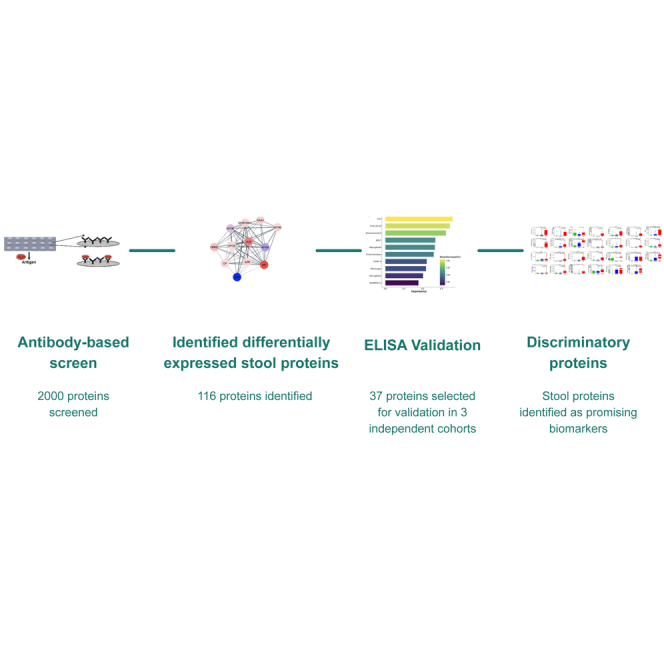

## In Brief

The holy grail in colon cancer biomarker research is to identify biomarkers that can accurately detect early precancerous lesions, namely advanced adenomas. Here, we report several stool biomarkers that detect advanced adenomas with significantly improved accuracy, with stool fibrinogen topping the list with a diagnostic accuracy of 86%, easily eclipsing the diagnostic performance of current test. Further validation of these novel biomarkers in additional patient cohorts as well as in randomized clinical trials could significantly aid in early detection of CRC and better treatment for this leading cancer.

In the United States, colorectal cancer (CRC) is the third most common cause of cancer and is the second leading cause of cancer death for both men and women (1). However, CRC can be significantly prevented with early detection through effective screening (2). While there are many different CRC screening options, only 68% of recommended patients were screened in 2018 (https://www.cdc.gov/colorectal-cancer/statistics/index.html). Since the survival rate for CRC improves from 64% to 90% if detected at an early stage, it is necessary to increase CRC screening based on established guidelines from the US Preventive Services Task Force (3, 4).

Randomized controlled trials demonstrate that colonoscopy reduced CRC mortality by 67%, whereas annual fecal occult blood testing (FOBT, *i.e.*, guaiac FOBT) reduced CRC mortality by 32% and biennial FOBT reduced CRC mortality by 18% (5, 6, 7). However, FOBT has a high number of false positives and negatives; therefore, FOBT should not be used alone (8). In a large, randomized trial, the sensitivity of the fecal immunochemical test (FIT) for detecting CRC was 73.8% with a sensitivity of only 23.8% for advanced precancerous lesions (9). In the largest studies, FIT-DNA (Cologuard) was more sensitive for detecting CRC and advanced adenomas than FIT but detected ∼42.4% of the precancerous lesions (while FIT detected only 23.8%) (10). FIT-DNA was less specific than FIT, with significantly more false-positive results, and significantly more expensive (9, 10, 11).

Colonoscopy is regarded as the screening test of choice in the United States, but even it has disadvantages, including possibly missing key lesions, its invasive nature, a 1:1000 perforation rate, required sedation, and a high mean cost of >$2000 (6, 12, 13). Thus, screening tests for CRC and advanced adenomas need to be significantly improved.

When evaluated against direct visualization tests and endoscopy, stool tests are simple, inexpensive, and widely available for CRC screening (14). Both guaiac FOBT and FIT detect blood in stool, but guaiac FOBT requires a significant amount of heme to cause a visible color change. Therefore, any dietary changes could produce a false positive or negative, resulting in FOBT detection of hemoglobin to be insensitive and nonspecific (15). Some studies have mined potential biomarkers utilizing nontargeted mass spectrometry, but high-abundance proteins obscure low-abundance proteins (16, 17). Other studies have targeted certain proteins as possible diagnostic CRC markers based on known functions of the protein (18). Few comprehensive screens of CRC stool have been carried out until recently. The largest proteomic screen of stool proteins in CRC in the literature was conducted by Li *et al*. (19), utilizing an aptamer-based screen to screen ∼1000 proteins, resulting in the identification of several stool biomarkers for CRC, including MMP-9, fibrinogen, myeloperoxidase (MPO), and haptoglobin.

Here, we report the largest targeted proteomic screen of CRC stools ever reported, interrogating 2000 proteins to identify target proteins without compromising the detection of low-abundance proteins. By using an antibody microarray, antibodies capture their specific protein, and a signal is detected by fluorescence (20, 21). The current work utilizes the largest antibody-based proteomic platform currently reported and represents the first application of this novel platform toward stool biomarker discovery.

In this study, multiple stool proteins were identified as diagnostic biomarkers of CRC utilizing a novel 2000-plex antibody–based immunoproteomic screen, and subsequently platform-validated by ELISA in an independent patient cohort. The reported stool protein biomarkers exhibit superior diagnostic metrics compared with current biomarkers in identifying CRC. Furthermore, we also report potential stool biomarkers that may help identify advanced adenomas, an area that has substantial biomarker potential, particularly for the early detection of malignancy and screening of high-risk subjects.

## Experimental Procedures

### Patients, Sample Collection, and Sample Preparation

Included in the study were patients diagnosed with CRC or intestinal adenoma, and healthy controls (HCs). Stool samples were obtained from four cohorts, referred to as cohorts I to IV. Cohort I consisted of 12 patients with CRC and 12 HCs, all purchased from iProcess Global Research. Of the CRC subjects, the percentages of tumor/nodes/metastasis (TNM) stage 1, 2, 3, and 4 lesions were 50%, 16.7%, 16.7%, and 16.7%, respectively, with grade 2, 3, and 4 tumors constituting 16.7%, 66.7%, and 16.7%, respectively. Cohort II consisted of 40 patients with CRC, 15 with advanced intestinal adenoma (all being >2 cm), and 23 HCs, all from subjects seen at the Mayo Clinic (Rochester, MN). Of the CRC subjects, the percentages of TNM stage 1, 2, 3, and 4 lesions were 25%, 17.9%, 50%, and 7.1%, respectively, with grade 2, 3, and 4 tumors constituting 17.9%, 75%, and 7.1%, respectively. Patients in cohort II were Caucasians, whereas patients in cohort I were of Indian descent. Cohort III consisted of 14 CRC and 15 HC stool samples, all of Caucasian origin, purchased from Geneticist, whereas cohort IV consisted of 22 stool samples from CRC and 25 from HCs, all of Indian origin, purchased from iProcess Global Research. All subjects signed informed consent, and the study was approved by the ethics boards of the Mayo Clinic (IRB #16-003882) and the University of Houston (IRB #15192-EX), respectively. Relevant clinicopathological information was collected from all patients and HCs, including age, gender, TNM stage, tumor size, tumor number, tumor nodes, tumor site, tumor location, and tumor metastasis, as detailed in Supplemental Tables S1 and S2. The consort diagram in Supplemental Fig. S1 summarizes the study protocol, workflow, validation steps, and study cohorts.

CRC stool samples were obtained prior to any preoperative chemotherapy or radiotherapy. AA stool samples were from screening colonoscopies (hence, there is no prior treatment or diagnosis), collected as first movement before bowel preparation prior to the colonoscopy. Samples were collected in sterile containers, placed in ice boxes immediately, and stored at −80 °C within 1 h of collection. Samples were then extracted for stool protein using the following method. The frozen feces were thawed at room temperature, and a mass (M) of 100 mg feces was dissolved in 600 μl of protein extraction buffer (594 μl NP40 + 6 μl protease inhibitor). After the solution underwent 10 cycles of 2-min shaking and 1-min ice bath, it was centrifuged for 5 min at 4 °C at 3000 rpm. Following this, the supernatant was ice-baked for 60 min and centrifuged for 30 min at 4 °C at a rotation speed of 10,000 rpm. Afterward, the supernatant was once again extracted. The final volume (V) of the stool protein ranged from 0.45 to 0.6 ml and was recorded. The final concentration of the stool sample was normalized by dividing M by V (mg/ml).

### L2000 Antibody–Based Array Screening

The stool samples used for the antibody-based proteomic screen were from cohort I, which included 12 randomly selected CRC samples and 12 HC samples. After stool proteins were extracted, the proteins were interrogated using a 2000-plex antibody–based protein screening platform, L2000, a commercially available array (Raybiotech), bearing a library of 2000 antibodies specific for 2000 different proteins. These antibodies (and their targeted antigens) were not selected in any fashion but offer good coverage of human proteins belonging to all functional categories, with no bias toward intestinal, secreted, dietary, or microbial proteins.

Specifically, once the samples were prepared, they went through dialysis in separate dialysis tubes for 3 h at 4 °C with gentle stirring to facilitate the removal of small, unwanted compounds from macromolecules in the solution by passive diffusion through a semipermeable membrane. The dialysis step was repeated by exchanging the dialysis buffer (to prepare 1 l dialysis buffer of 0.2 g KCl, 8 g NaCl, 0.2 g KH_2_PO_4_, and 1.15 g Na_2_HPO_4_, which were dissolved in PBS, with pH = 8.0). The dialyzed samples were spun for 5 min at 10,000 rpm to remove any particulates or precipitates. The supernatants were transferred to a clean tube. To determine protein concentration, bicinchoninic acid assay kits were used. Samples were then biotinylated. For further dialysis of the biotinylated samples, the same dialysis step mentioned above was repeated. The samples were then ready for microarray analysis on prefabricated antibody-coated slides (catalog no.: AAH-BLG-2000-8). These slides were buffer equilibrated and blocked, followed by the addition of biotin-labeled test samples on the array slides, followed by the addition of fluorescence-tagged streptavidin, and then scanned using a GenePix 4000B Scanner.

### ELISA-Based Cross-Sectional Validation

Based on hits from the antibody-based screen, protein markers were identified and then validated in an independent validation cohort (cohort II) using ELISA. Cohort II consisted of 40 patients with CRC, 15 with intestinal adenoma, and 23 HCs, all from the Mayo Clinic. Promising biomarker proteins were further validated in cohorts III and IV, as outlined in Supplemental Fig. S1. ELISA was performed utilizing commercial kits following the manufacturer’s instructions while the operator was blinded throughout the procedure. The ELISA procedure involved adding a diluted stool protein extract to a 96-well plate that was precoated with capture antibodies. Following sample incubation, the detection antibody was added, and then streptavidin–horseradish peroxidase and finally, the substrate was added. A microplate reader (ELX808 from BioTek Instruments) was then used to read the absorbance at 450 nm. The values obtained from the microplate reader were compared with a standard curve within each ELISA plate to interpolate the absolute expression level of stool protein biomarkers in each test sample. Concentrations were converted into picograms per milliliter and normalized by the stool mass; therefore, the final mass-normalized concentration unit was picograms per milligram for each stool biomarker. The supplier and optimal dilution factor used for each stool protein ELISA kit can be found in Supplemental Table S3.

### Statistical Analysis

The processing of stool protein biomarker data and related analyses were conducted using GraphPad Prism 7 (GraphPad), Microsoft Excel, and R (version 3.6.2). Since the biomarker data were non-normally distributed, comparisons between groups were analyzed using the Mann–Whitney *U* test. *q* Values (*p* values adjusted for false discovery rate, for multiple testing correction) were also computed for each biomarker. Receiver operating characteristic (ROC) curve, area under the curve (AUC), and the corresponding sensitivity, specificity, and cutoff values (pictograms per milligram) were obtained using the pROC package in R. Principal component analysis was conducted to explore the underlying relationships among the various proteins tested comparing CRC to HC, adenoma to HC, and CRC to adenoma, with the possibility of data reduction.

Values below the detection limit (initially recorded as 0) were replaced by the 10% value of the smallest nonzero value of each protein. The levels of each protein were then standardized to have a mean of zero and a unit standard deviation, after applying log_2_ transformation. The age of the patients was also transformed and standardized in the same manner.

To identify the most discriminatory proteins that distinguished the study groups (control *versus* CRC, control *versus* adenoma, and CRC *versus* adenoma), we used a logistic regression model with least absolute shrinkage and selection operator using *glmnet* package in R (version 4.0.3). The value of the tuning parameter λ, which controls the overall strength of the penalty, was selected using leave-one-out crossvalidation and choosing the value that gives the most regularized model such that the mean squared error is within one standard error of the minimum (usually denoted by *lambda.1se*). Considering that the sample size is relatively small, we added a bootstrap procedure in the analysis to obtain optimism-corrected performance metrics (22). (For details, see Section 5.3.4 of *Clinical prediction models* (23). Here, performance metrics included AUC, prediction accuracy, Brier scores, sensitivity, and specificity. All regression models reported in this analysis were adjusted for subjects’ age and gender.

Associations between tumor characteristics and each of the stool biomarkers from the generated models were evaluated using ANOVA. Separate ANOVAs were computed with each biomarker as the outcome and various clinicopathological features (tumor site, grade, size, depth, and TNM stage) as independent variables.

### Heatmap, Cluster Analysis, Random Forest Classification, Gene Ontology, Kyoto Encyclopedia of Genes and Genomes Pathway, Cytoscape and Reactome Pathway Mapping, and Public Database Searches

Data from the antibody-based screen were used to generate a heatmap and to perform cluster analysis. In the heatmap, if the fold change (FC) of the protein was greater than 2 and the *p* value was less than 0.05, it was considered to be significantly increased. Cluster analysis brings together proteins with similar expression patterns and clusters them in an unsupervised manner. Random forest, a machine-learning algorithm for dimension reduction, was utilized with 1000 bootstrap sampling iterations. The relative importance of each biomarker in disease classification was then ranked using the GINI index. The aforementioned analyses were run using the sklearn.ensembl R package. By searching the public databases Firebrowse, Oncomine, The Cancer Genome Atlas, and The Human Protein Atlas, the expression of CRC-associated stool protein biomarkers at the RNA and protein levels, as documented in the previous literature, was ascertained.

Gene Ontology (GO) analysis was plotted for the top 10 proteins in distinguishing CRC *versus* HC. Molecular Function, Biological Process, and Kyoto Encyclopedia of Genes and Genomes pathways were plotted for molecules with a significant *p* value (*p* < 0.05) and in the order of fold enrichment. Protein–protein interaction networks were created for the top 116 proteins with *p* < 0.05 through the Cytoscape stringApp with a confidence cutoff of 0.4. MODE clustering was performed, and the largest two clusters are shown. Reactome pathway analysis of the dysregulated proteins indicated over-representation of selected functional pathways, as indicated.

## Results

### 2000-Plex Antibody–Based Screening of CRC Stools

Proteins from the stool samples of cohort I (12 CRCs, 12 HCs) were extracted and interrogated using a 2000-plex antibody–based protein screening platform, L2000. Of 2000 proteins assayed using the L2000 screen, 116 proteins exhibited *p* < 0.05, comparing CRC to HC. Of these 116 proteins, 45 proteins expressed FC >2 in CRC when compared with HC, with only one protein exhibiting *q* < 0.05 (*p* values adjusted for false discovery rate, after multiple testing correction), namely stool haptoglobin.

**Fig. 1:**
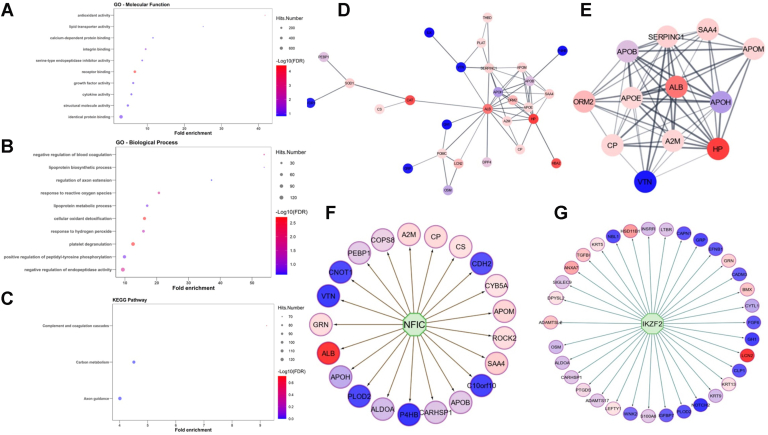
**Functional pathway enrichment analysis of molecules differentially expresses in CRC *versus* HC stool samples in cohort I using Gene Ontology, biological processes, KEGG pathway, and protein–protein interaction networks.** Of 2000 proteins assayed using the L2000 screen, 116 proteins were significantly different between CRC and HC stools in cohort I. Of the 116 proteins, 45 proteins expressed FC > 2 in CRC when compared with HC. *A*–*C*, Gene Ontology analysis was plotted for the top 10 proteins in CRC *versus* HC stool. Molecular function, biological process, and KEGG pathways are plotted for molecules with significant *p* value (*p* < 0.05) and in the order of fold enrichment. The magnitude of the *dots* represents the total number of genes fitting to the annotation term, and the color of the dots is illustrative of –log_10_FDR value. *D* and *E*, protein–protein interaction networks were created for the top 116 proteins with *p* < 0.05 (CRC *versus* HC in cohort I) using the Cytoscape stringApp with a confidence cutoff of 0.4. MODE clustering was performed, and the two largest clusters are shown. The color of each node corresponds to the FC. Nodes with an FC less than two range in color from *blue* to *purple*, whereas those with an FC greater than two range from *pink* to *red*. The confidence score of each interaction is displayed as the edge thickness and opacity. Reactome pathway analysis of the dysregulated proteins indicate over-representation of the following functional pathways: post-translational protein phosphorylation, binding and uptake of ligands by scavenger receptors, regulation of insulin-like growth factor (IGF) transport, hemostasis, post-translational protein phosphorylation, and platelet degranulation in CRC. *F* and *G*, the top transcription factor regulator (*F*) and top signaling molecule regulator (*G*) identified for the top 116 proteins with *p* < 0.05 (CRC *versus* HC in cohort I) are shown, identified using the iRegulon plugin. Nodes with an FC less than two range in color from *blue* to *purple*, whereas those with an FC greater than two range from *pink* to *red*. CRC, colorectal cancer; FC, fold change; FDR, false discovery rate; HC, healthy control; KEGG, Kyoto Encyclopedia of Genes and Genomes.

GO analysis of the proteins dysregulated in CRC stool revealed a significant over-representation of molecular functions related to antioxidant activity, integrin/receptor binding, cytokines, and serine type endopeptidase activity and biological processes related to blood coagulation, antioxidants, and lipoprotein biosynthesis in patients with CRC compared with HC (Fig. 1, *A* and *B*). Proteins related to complement/coagulation cascades and carbon metabolism were also dysregulated in CRC (Fig. 1*C*). Protein–protein interaction networks of importance for the top 116 proteins implicated hemostasis, post-translational protein phosphorylation, platelet degranulation, regulation of insulin-like growth factor transport and uptake by insulin-like growth factor binding proteins, and plasma lipoprotein assembly pathways (Fig. 1, *D* and *E*). The protein–protein interaction network with MCODE clustering is shown in Figure 1*E*. Of the top 116 dysregulated proteins, the top transcription factor regulator identified was nuclear factor IC (NFIC) (Fig. 1*F*). Of the top 116 proteins, the top signaling molecule regulator identified was IKZF2 (Fig. 1*G*).

### Array-Based Screening of CRC Stool Samples for 2000 Proteins

**Fig. 2:**
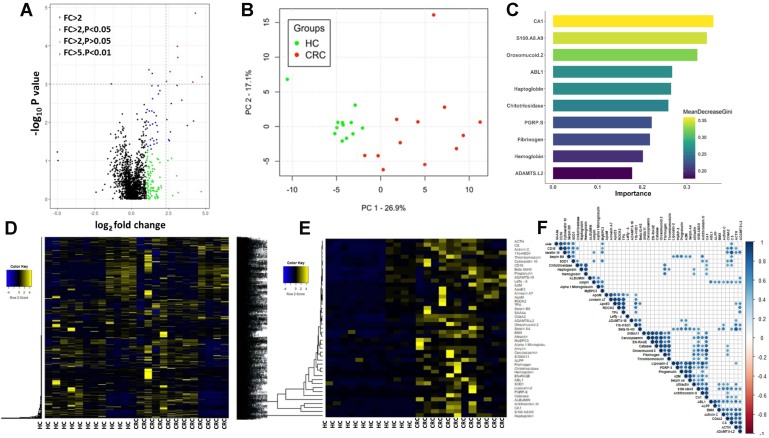
**Antibody array–based proteomic screening of CRC stool and graphical representation of significantly different proteins in CRC *versus* HC.***A*, a volcano plot representation of results from the antibody array–based screening of 2000 proteins analyzed in 24 stool samples (12 CRCs and 12 HCs) is plotted using R Studio. Log transformed fold change (FC) and negative log _10_*p* value are plotted on *x*- and *y*-axis, respectively. Of the 116 proteins that exhibited Mann–Whitney *p* value <0.05, 45 proteins were elevated at FC >2 in CRC stool when compared with HC stool. Each *dot* represents one of the 2000 proteins. *B*, a 3D principle component analysis (PCA) plot of all subjects, using the 116 proteins that were differentially expressed (CRC *versus* HC, *p* < 0.05). CRC is represented by a *red circle*, whereas HC is represented by a *green circle*. *C*, random forest analysis was performed using the top 45 proteins (CRC *versus* HC, Mann–Whitney *p* value <0.05, FC >2) to identify proteins that best distinguished CRC from HC. Plotted are the 10 most discriminatory stool proteins based on their differentiating capacity. These 10 proteins are ordered by their GINI coefficient (importance in discrimination). *D* and *E*, show the heat map representation of all 2000 proteins and the top 45 proteins (*p* < 0.05; FC >2) elevated in CRC stool when compared with HC stool, respectively. Proteins expressed at the median level are shown in *black*, proteins whose expression levels are above the median are shown in *blue*, whereas proteins whose expression is below the median are shown in *yellow*. R Studio was used to plot the heatmap. A hierarchical clustering algorithm was used. *F*, shows the correlation matrix of the top 45 upregulated proteins in CRC *versus* HC stool in cohort I. Pearson’s correlation coefficient was calculated correlation between each pair. Hierarchical clustering is used to plot the correlation profiles using R Studio. Each *circle* signifies the correlation between two proteins. *Blue* indicates positive correlation, whereas *red* indicates negative correlation, as indicated by the color bar. CRC, colorectal cancer; HC, healthy control.

The overall results of the initial antibody array–based “omics” screen of 2000 proteins are shown in Figure 2*A* as a Volcano plot with each dot representing one of the 2000 proteins interrogated. When compared with HC stool, 45 proteins were found to be elevated in CRC stool (*p* < 0.05, FC >2). The top 116 proteins that were significantly different (*p* < 0.05) underwent principal component analysis, and the first two principal components explained 72.4% of the variance (Fig. 2*B*). The use of a machine learning algorithm, random forest analysis, identified carbonic anhydrase 1, haptoglobin, Peptidoglycan Recognition Protein-S (PGRP-S), Orosomuccoid2, ADAMTS.L2, ABL1, S100 A8A9, Serpin A4 (kallistatin), catalase, and chitotriosidase as the top 10 most discriminatory stool proteins that distinguished CRC from HC (Fig. 2*C*). Protein expression profiles from the L2000-based screen were used to generate a heatmap, which grouped proteins with similar expression patterns together, as shown in Figure 2*D*. In addition, the top 45 proteins elevated in CRC stool when compared with healthy stool (*p* < 0.05, FC >2) are shown as a heatmap in Figure 2*E*. Correlation analysis of these 45 proteins revealed 8 to 10 discrete protein clusters with similar expression profiles, as shown in Figure 2*F*.

Interestingly, seven proteins were significantly reduced in CRC stool, namely, DMRN9, GSTP1, HGH, HMGB3, ILK, NOTCH-2, and P4HB. As no common biological pathway was implicated by these reduced proteins, they were not pursued further.

### ELISA Validation of Elevated Stool Proteins in CRC Using Cohort II

**Fig. 3:**
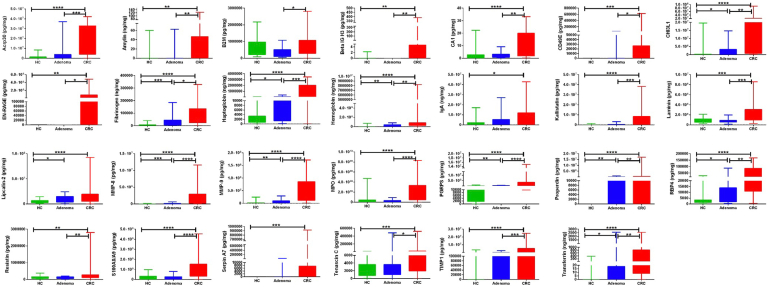
**Validating the expression profiles of 27 proteins in CRC, advanced adenoma, and HC stool using ELISA in CRC cohort II.** Twenty-seven proteins were validated by ELISA in CRC, advanced adenoma, and HC stool protein extracts from the Mayo cohort (cohort II) in 78 subjects (CRC = 40; adenoma = 15; and HC = 23). The expression of proteins in all subjects is represented as box plots, and statistical significance is shown as follows (∗*p* < 0.05;∗∗*p* < 0.01;∗∗∗*p* < 0.001; and ∗∗∗∗*p* < 0.0001). *Green*, *blue*, and *red colors* are used to represent HC, adenoma, and CRC, respectively, in box plots. CRC, colorectal cancer; HC, healthy control.

Based on the correlation clustering (Fig. 2*F*) and random forest analysis (Fig. 2*C*) of the stool proteins identified using the antibody-based screen, 37 proteins were selected for ELISA validation, which represents a platform that is orthogonal to the one used for the initial screen. The selected proteins, ELISA manufacturer, stool dilution, reason for protein selection, and outcome of the ELISA are listed in Supplemental Table S3. Of the 37 proteins initially selected, 27 proteins were advanced for validation in the first independent validation cohort, cohort II (40 CRCs, 15 adenomas, 23 HCs), based on preliminary ELISA results. These proteins included ACRP30 (adiponectin), amylin, B2M, beta IG-H3, carbonic anhydrase 1, integrin a5 (CD49E), YKL-40 (CHI3L1), S100A12 (EN-RAGE), fibrinogen, haptoglobin, hemoglobin, IgA, Serpin A4 (kallistatin), laminin, Lipcallin-2, MMP-8, MMP-9, MPO, PGRP-S, properdin, RBP4, resistin, S100A8A9, Serpin A7 (TBG), Tenascin C, TIMP-1, and transferrin. The ELISA validation results are shown in Figure 3, normalized by stool mass. Of the 27 stool proteins tested by ELISA, 26 proteins were significantly higher in the CRC stool than in the HC stool. Among them, 10 proteins, including YKL-40 (CHI3L1), fibrinogen, haptoglobin, hemoglobin, MMP-8, MMP-9, PGRP-S, properdin, RBP4, and transferrin, showed an increasing trend from the HC group to the adenoma group to the CRC group, with significant differences being noted between each successive group. Stool IgA and Serpin A7 (TBG) levels were only statistically different between the HC group and the CRC group.

ROC analysis was next performed using the ELISA data, for discriminating CRC from HC, as displayed in Table 1. The sensitivity, specificity, and cutoff values for each protein are also shown in this table. MMP-8 (AUC = 0.95), MMP-9 (AUC = 0.92), hemoglobin (AUC = 0.92), PGRP-S (AUC = 0.92), and haptoglobin (AUC = 0.91) were the top 5 stool proteins with the highest accuracy values for distinguishing CRC from HC (AUC >0.90) in cohort II, with ACRP30 (adiponectin) being close behind (AUC = 0.90). Of these proteins, stool MMP-8 and MMP-9 exhibited the highest sensitivity (≥0.90), whereas several stool proteins exhibited perfect specificity values for the diagnosis of CRC.

**Table 1:** Protein levels of 27 stool proteins in CRC, adenoma, and healthy subjects, as validated by ELISA in cohort II

Protein	Stool protein, pg/mg, mean (median)			FC			Comparison of CRC *versus* HC			
	CRC Mean (median)	HC Mean (median)	Adenoma Mean (median)	CRC/HC	CRC/adenoma	Adenoma/HC	Cutoff	AUC	Sensitivity	Specificity
Acrp30 (adiponectin)	17,259,011 (9,634,282)	1,260,139 (398,029)	6,311,859 (625,564)	13.7	2.7	5.0	1,902,318	0.90∗∗∗∗	0.85	0.87
Amylin	28 (14)	3 (0)	6 (0)	9.4	4.6	2.0	9	0.72∗∗∗∗	0.55	0.96
B2M	877,392 (714,566)	641,816 (492,200)	366,852 (316,122)	1.4	2.4	0.6	709,073	0.58	0.53	0.74
Beta IG-H3	19 (0)	0 (0)	0 (0)	115.6	>>	0.0	0	0.67∗∗∗	0.43	0.91
Carbonic anhydrase 1	11 (8)	2 (1)	3 (2)	5.0	4.4	1.1	4	0.8∗∗∗∗	0.65	0.96
Fibrinogen	92,578 (74,836)	7211 (3049)	41,733 (36,955)	12.8	2.2	5.8	17,673	0.89∗∗∗∗	0.85	0.91
Haptoglobin	883,949 (231,923)	4100 (1321)	52,620 (3655)	215.6	16.8	12.8	34,260	0.91∗∗∗∗	0.78	1.00
Hemoglobin	726,211 (574,300)	42,062 (1735)	205,392 (57,155)	17.3	3.5	4.9	171,206	0.92∗∗∗∗	0.83	0.96
IgA	7,323,995 (2,512,175)	1,998,991 (264,160)	4,762,974 (2,264,416)	3.7	1.5	2.4	2,659,267	0.69∗∗	0.50	0.83
Integrin a5 (CD49e)	52,102 (0)	0 (0)	3367 (0)	>>	15.5	>>	965	0.7∗∗∗∗	0.40	1.00
Laminin	2,163,461 (1,587,352)	901,611 (838,922)	840,237 (766,396)	2.4	2.6	0.9	1,495,522	0.78∗∗∗∗	0.55	0.91
Lipocalin 2	17,870,354 (9,359,314)	4,901,538 (3,535,274)	9,571,034 (8,015,725)	3.6	1.9	2.0	7,229,135	0.8∗∗∗∗	0.73	0.83
MMP-8	2,090,420 (975,703)	10,524 (0)	99,374 (49,134)	198.6	21.0	9.4	83,899	0.95∗∗∗∗	0.90	0.96
MMP-9	47,138,718 (35,498,235)	2,500,668 (1,543,843)	6,926,037 (299,818)	18.9	6.8	2.8	4,190,831	0.92∗∗∗∗	0.93	0.96
MPO	2,295,297,698 (1,441,910,454)	483,089,795 (212,444,218)	259,204,858 (199,591,492)	4.8	8.9	0.5	310,532,890	0.84∗∗∗∗	0.88	0.70
PGRP-S	2,156,302 (859,425)	84,292 (30,498)	170,842 (90,801)	25.6	12.6	2.0	171,731	0.92∗∗∗∗	0.85	0.91
Properdin	1,193,252 (185,464)	0 (0)	98,473 (0)	>>	12.1	>>	1	0.88∗∗∗∗	0.75	1.00
RBP4	53,214 (36,582)	4634 (2284)	16,497 (8310)	11.5	3.2	3.6	7307	0.89∗∗∗∗	0.85	0.91
Resistin	373,347 (168,681)	117,677 (93,149)	105,111 (111,184)	3.2	3.6	0.9	163,152	0.73∗∗∗	0.63	0.83
S100A12 (EN-RAGE)	545,921 (0)	0 (0)	0 (0)	>>	>>	>>	461,049	0.65∗∗∗∗	0.30	1.00
S100A8,A9	1,082,758,215 (639,112,650)	169,688,206 (0)	191,435,993 (191,465,482)	6.4	5.7	1.1	437,157,100	0.85∗∗∗∗	0.68	0.91
Serpin A4 (kallistatin)	5,729,368 (3,156,369)	72,951 (0)	439,336 (0)	78.5	13.0	6.0	1	0.86∗∗∗∗	0.75	0.91
Serpin A7 (TBG)	38,486 (0)	0 (0)	38,486 (0)	>>	16.9	>>	1	0.71∗∗∗∗	0.43	1.00
Tenascin C	40,722 (5831)	2169 (1384)	40,722 (2169)	18.8	1.2	15.9	4672	0.76∗∗∗∗	0.60	0.91
TIMP-1	4,046,174 (2,423,325)	147,359 (0)	4,046,174 (147,359)	27.5	10.0	2.8	224,688	0.83∗∗∗∗	0.70	0.96
Transferrin	529 (235)	16 (0)	529 (16)	32.1	3.0	10.7	8	0.87∗∗∗∗	0.78	0.96
YKL-40 (CHI3L1)	13,626,252 (5,456,786)	1,060,034 (186,416)	2,273,428 (747,833)	12.9	6.0	2.1	784,442	0.88∗∗∗∗	0.80	0.96

The validation cohort of 78 subjects was comprised of 40 CRC patients, 15 adenoma patients, and 23 HCs. Mann Whitney U test *p*-values are denoted using asterisks (∗*p* < 0.05; ∗∗*p* < 0.01; ∗∗∗*p* < 0.001, ∗∗∗∗*p* < 0.0001).

ROC analysis was also performed to assess the ability of these stool proteins to discriminate advanced adenoma from HC in cohort II, as displayed in Table 2. Stool fibrinogen (AUC = 0.86), MMP-9 (AUC = 0.8), hemoglobin (AUC = 0.8), MMP-8 (AUC = 0.79), and PGRP-S (AUC = 0.78) were the top 5 stool proteins with the highest discriminatory potential for distinguishing advanced adenoma from HC, with stool fibrinogen topping the list with an accuracy value of 0.86, whereas the others exhibited ROC AUC values of 0.80 or below. Indeed, stool fibrinogen had a sensitivity value of 0.93, surpassed only by stool PGRP-S (sensitivity = 1.0).

**Table 2:** Discriminatory capacity of 27 stool proteins in distinguishing colorectal adenoma from HCs, based on ELISA in cohort II

Protein	Cutoff	AUC	Sensitivity	Sensitivity^90^	Specificity
Acrp30 (adiponectin)	2,219,393	0.5	0.33	0.30	0.87
Amylin	8	0.51	0.20	0.20	0.96
B2M	48,006	0.35	1.00	0.00	0.00
Beta IG-H3	0	0.43	1.00	0.00	0.00
Carbonic anhydrase 1	1	0.61	0.80	0.20	0.44
Fibrinogen	4205	0.86^d^	0.93	0.54	0.65
Haptoglobin	3447	0.71^a^	0.60	0.32	0.83
Hemoglobin	11,402	0.8^b^	0.80	0.53	0.78
IgA	238,122	0.67	0.87	0.20	0.48
Integrin α5 (CD49e)	50,504	0.53	0.07	0.20	1.00
Laminin	488,531	0.45	0.93	0.13	0.22
Lipocalin 2	5,199,180	0.72^a^	0.73	0.40	0.70
MMP-8	8190	0.79^b^	0.67	0.56	0.87
MMP-9	2,107,885	0.8^b^	0.80	0.60	0.83
MPO	145,101,494	0.48	0.73	0.00	0.48
PGRP-S	30,834	0.78^b^	1.00	0.27	0.57
Properdin	56,761	0.67^c^	0.33	0.40	1.00
RBP4	5726	0.73^a^	0.67	0.17	0.91
Resistin	111,184	0.49	0.53	0.00	0.57
S100A12 (EN-RAGE)	0	0.5	1.00		0.00
S100A8, A9	97,557,056	0.55	0.67	0.07	0.52
Serpin A4 (kallistatin)	422,747	0.6	0.27	0.28	0.96
Serpin A7 (TBG)	6404	0.57	0.13	0.22	1.00
Tenascin C	808	0.58	0.93	0.17	0.35
TIMP-1	122,894	0.61	0.27	0.30	0.96
Transferrin	10	0.64^a^	0.33	0.39	0.96
YKL-40 (CHI3L1)	747,833	0.71^a^	0.53	0.53	0.96

Sensitivity^90^: Sensitivity at 90% specificity.

a *p* < 0.05.

c *p* < 0.01.

b *p* < 0.001.

d *p* < 0.0001.

ROC analysis was also performed for discriminating CRC from advanced adenoma, as displayed in Supplemental Table S4. MPO (AUC = 0.88), MMP-8 (AUC = 0.87), PGRP-S (AUC = 0.85), MMP-9 (AUC = 0.84), and S100A8A9 (AUC = 0.83) were the top 5 stool proteins with the highest discriminatory potential for distinguishing advanced adenoma from CRC in cohort II, with the best two performers being MPO and MMP-8. Of these stool proteins, MPO and PGRP-S exhibited the highest sensitivity for discriminating CRC from adenoma.

**Fig. 4:**
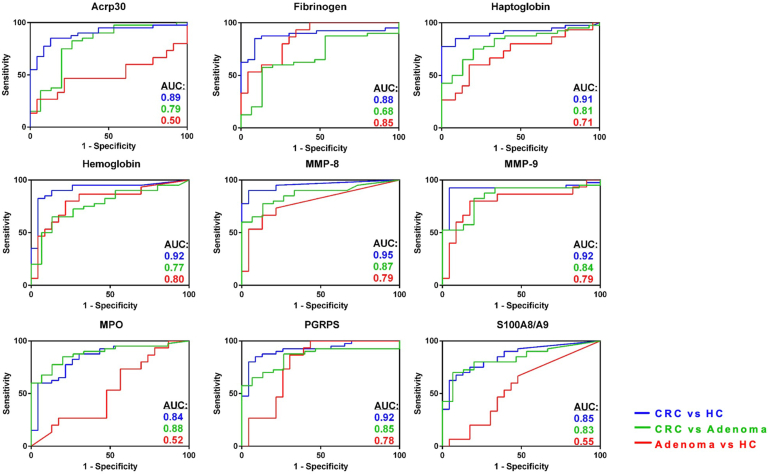
**Evaluating the discriminatory potential of nine proteins using receiver operative curve (ROC) analysis in CRC cohort II.** Area under the curve (AUC) plots are shown for the top 9 proteins validated by ELISA in cohort II (CRC = 40; advanced adenoma = 15; and HC = 23) for all three disease comparisons, CRC *versus* HC (*blue*); CRC *versus* adenoma (*green*); and adenoma *versus* HC (*red*). CRC, colorectal cancer; HC, healthy control.

Collectively, based on ROC AUC analysis, the top 9 stool proteins for distinguishing the subject groups (*i.e.*, CRC *versus* HC, adenoma *versus* HC, or CRC *versus* adenoma) were ACRP30 (adiponectin), fibrinogen, haptoglobin, hemoglobin, MMP-8, MMP-9, MPO, PGRP-S, and S100A8A9, as plotted in Figure 4. Interestingly, several stool proteins were significantly higher in more advanced stages of CRC, including B2M, transferrin, MMP-8, MMP-9, TMP1, IgA, amylin, and beta IG-H3 (Supplemental Fig. S2). Perhaps more importantly, we also assessed if early stage CRC can be distinguished from healthy stools by these stool biomarkers. Indeed, all these stool proteins exhibited significant increases in stage 1 and 2 CRC compared with HC: fibrinogen (FC = 11.1; *p* < 0.0001), MMP-8 (FC = 185.8; *p* < 0.0001), MMP-9 (FC = 14.4; *p* < 0.0001), MPO (FC = 4.2; *p* < 0.0003), PGRP-S (FC = 19.6; *p* < 0.0001), haptoglobin (FC = 160.5; *p* < 0.0001), and hemoglobin (FC = 10.6; *p* < 0.0001), alluding to the potential utility of these stool proteins in early diagnosis.

### Identification of Multimarker Stool Protein Panels, After Adjustment for Demographics

All the aforementioned analyses were carried out focusing on the performance of each biomarker in isolation, without factoring in the impact of other biomarkers or demographic confounders. Next, we examined the performance of biomarker combinations after adjusting for age and gender. A logistic regression with least absolute shrinkage and selection operator regularization was used to assess the ability of the different stool proteins to distinguish differences between the subject groups. The top 5 most discriminatory proteins in each panel are listed in Supplemental Table S5. Performance was evaluated using AUC, prediction accuracy, Brier score, sensitivity, and specificity. After adjustment for age and gender, the five topmost discriminatory proteins that distinguished CRC *versus* HC, as a panel, were MMP-8, hemoglobin, Serpin A4 (kallistatin), fibrinogen, and properdin, with an AUC of 0.97. This panel overlaps with the single marker results reported in Table 1, as MMP-8 (AUC = 0.95) and hemoglobin (AUC = 0.92) had the highest AUC scores. This panel of stool proteins provides an improved AUC compared with single markers.

For distinguishing HC *versus* advanced adenoma, the top 5 proteins were stool hemoglobin, fibrinogen, MMP-8, properdin, and PGRP-S, with a panel AUC of 0.81, after adjusting for age and gender. This panel overlaps with the single marker results reported in Table 2, as fibrinogen (AUC = 0.86), hemoglobin (AUC = 0.8), MMP-8 (AUC = 0.79), and PGRP-S (AUC = 0.78) had the highest AUC scores in Table 2. The ability of several stool proteins to distinguish advanced adenoma stool from healthy stool is noteworthy, given that most current screening tests fail to do so. For distinguishing CRC *versus* advanced adenoma, the top 3 proteins were stool ACRP30 (adiponectin), Serpin A4 (kallistatin), and S100 A8A9, with a panel AUC of 0.83, after adjusting for age and gender, as summarized in Supplemental Table S5.

We also examined how these stool biomarkers performed in distinguishing advanced neoplasia (CRC and AA combined) from HC stools. As detailed in Supplemental Table S6, the ROC AUC values of these proteins in distinguishing advanced neoplasia were intermediate between the ROC AUC values for distinguishing CRC alone or AA alone from healthy stools, as reported above in Tables 1 and 2. Several stool proteins exhibited excellent potential to identify advanced neoplasia (ROC AUC values of 0.85 or higher), including fibrinogen, MMP-8, MMP-9, haptoglobin, hemoglobin, and PGRP-S.

### Further ELISA Validation of Identified Stool Biomarkers in Two Additional Patient Cohorts

**Fig. 5:**
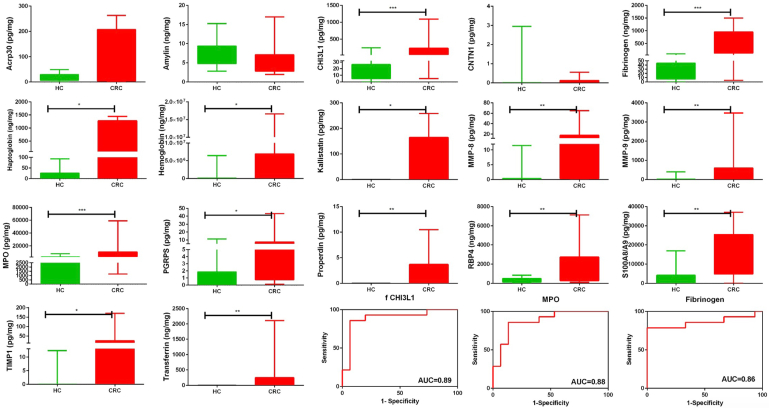
**Further ELISA validations of 17 proteins in CRC cohort III stool.** Seventeen selected proteins were further validated in CRC and HC stool from cohort III, comprised of Caucasian subjects, using ELISA. For these 17 stool proteins, based on AUC value, ROC AUC curves are also plotted for the top 3 most discriminatory proteins (CRC *versus* HC). AUC, area under the curve; CRC, colorectal cancer; HC, healthy control; ROC, receiver operating characteristic.

**Fig. 6:**
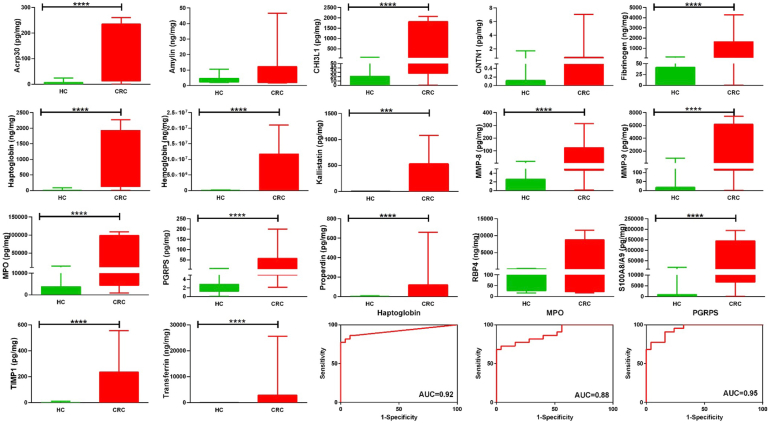
**Further ELISA validations of 17 proteins in cohort IV stool.** Seventeen selected proteins were further validated in CRC and HC stool from cohort IV, comprised of Indian subjects, using ELISA. For these 17 stool proteins, based on AUC value, ROC AUC curves are also plotted for the top 3 most discriminatory proteins (CRC *versus* HC). AUC, area under the curve; CRC, colorectal cancer; HC, healthy control; ROC, receiver operating characteristic.

Following the initial ELISA validation in cohort II, promising biomarkers were further validated in cohort III and cohort IV. Cohort III consisted of individuals of Caucasian ethnicity. Cohort IV consisted of individuals of Indian ethnicity. Both these cohorts were composed of CRC patients and HC but not adenoma patients. Seventeen leading biomarkers selected from the first validation study (in cohort II) were further validated in cohort III by ELISA, including ACRP30 (adiponectin), amylin, YKL-40 (CHI3L1), CNTN1, fibrinogen, haptoglobin, hemoglobin, Serpin A4 (kallistatin), MMP-8, MMP-9, MPO, PGRP-S, properdin, RBP4, S100A8A9, TMP-1, and transferrin. All five proteins that performed best in cohort II in distinguishing CRC were also significantly elevated in CRC stool in cohort III, including MMP-8, MMP-9, hemoglobin, PGRP-S, and haptoglobin (Fig. 5). In this cohort, YKL-40 (CHI3L1), MPO, and fibrinogen emerged as the top 3 proteins for discriminating CRC from HC, based on ROC AUC values. The same 17 proteins were also validated by ELISA in an additional cohort, cohort IV. All five proteins that performed best in cohorts II and III also significantly elevated in CRC stool in cohort IV, including MMP-8, MMP-9, hemoglobin, PGRP-S, and haptoglobin (Fig. 6). In this cohort, haptoglobin, MPO, and PGRP-S emerged as the top 3 proteins for discriminating CRC from HC, based on ROC AUC values (Fig. 6). Taking the ELISA validation data from all three cohorts, stool MMP-8, MMP-9, hemoglobin, PGRP-S, haptoglobin, MPO, and fibrinogen emerge as the most discriminatory stool proteins for distinguishing CRC from HCs, across cohorts and ethnicities. Finally, we combined subjects from all three validation cohorts (cohorts II + III + IV) and reanalyzed the performance of the stool biomarkers. The discriminatory performance of the stool biomarkers in the combined dataset was comparable to their performance in the individual datasets (comparing Supplemental Tables S6 and S7).

## Discussion

FOBT and FIT are the two widely used stool tests for detecting CRC, and both are based on detecting hemoglobin derivatives. A meta-analysis of 31 studies found that FOBT has an AUC of 0.77 to 0.87, specificity of 0.77, and sensitivity of 0.60 (24, 25). In a meta-analysis of 12 studies, researchers found that FIT has an AUC of 0.93, specificity of 0.91, and sensitivity of 0.93 (26). Some of the drawbacks of using these tests include high false-negative results and poor sensitivity for benign polyps (27, 28, 29). Stool tests like FOBT mainly detect the heme component of hemoglobin. This necessitates avoidance of all heme-containing foods like red meat, certain medications like nonsteroidal anti-inflammatory drugs, and vitamin C because of the potential for false-positive or false-negative results (27, 28, 29). FIT is 80% sensitive for CRC detection and approximately 20% to 30% sensitive for advanced neoplasia detection. To enhance advanced adenoma detection, repeated applications of FIT are required (27, 28, 29). This challenges patient adherence to screening. Lack of adherence ultimately decreases the efficiency of FOBT and FIT for screening (27, 28, 29). Genetic and epigenetic markers for detecting adenomas or early invasive CRC are a rapidly emerging field, though not currently used in isolation for screening yet (30).

Few studies have reported a comprehensive screen of CRC stool for potential protein biomarkers. An aptamer-based screen of 1317 proteins in CRC stool identified stool MMP-9, fibrinogen, MPO, and haptoglobin as potential biomarkers of CRC (19). Through mass spectrometry analysis of 834 proteins from human stool, 29 potential biomarkers were identified, including HBB, HBA1, HP, RBP4, MPO, TF, S100A8, and S100A9, with 0.94 AUC, 71% sensitivity, and 95% specificity for the detection of CRC (16). To validate the proteins identified by proteomic analysis, they performed an antibody-based evaluation of four protein candidates and showed that they can be validated (16). A similar study was conducted for the early detection of high-risk adenomas and CRC (17). One protein panel consisting of HP, LAMP1, SYNE2, and ANXA6 was identified for the detection of high-risk adenomas with 53% sensitivity and 95% specificity, and two protein panels, one with HP and LRG1 and one with HP, LRG1, RBP4, and FN1, were identified for high-risk adenomas and CRC detection, with sensitivity of 66% and 62%, respectively, at a specificity of 95% (17). In a different colonoscopy-controlled study, validation of α-2-macroglobulin, calprotectin, C3 complement, hemoglobin, haptoglobin, hemopexin, lactotransferrin, MPO, and serpin family F member 2 strongly suggested the use of these biomarkers in a multitarget FIT (31).

The current work represents the first attempt to interrogate 2000 stool proteins utilizing a novel antibody-based screen for additional stool biomarkers in CRC. Functional pathway analysis using GO revealed a significant over-representation of pathways related to antioxidant activity, integrin/receptor binding, cytokines, blood coagulation, and lipoprotein biosynthesis in patients with CRC compared with HC. Literature reports provide evidence of oncogene-directed overexpression of Nfr2 antioxidant program promoting proliferation and tumorigenesis *in vivo* (32). Direct evidence suggests the expression of α_2_/β_1_ integrin and cytokines is higher in human CRC tumors and cell lines (33, 34). High levels of blood coagulation and low-density lipoprotein receptor expression are also associated with CRC, consistent with GO predictions (35, 36).

Interestingly, NFIC and IKZF2 were identified as key regulators of the molecular cascades over-represented in CRC. NFIC is a transcription factor that has been reported as one of the key regulators of esophageal squamous cell carcinoma and gastric cancer proliferation and metastasis (37, 38, 39). Differentially expressed genes analysis revealed upregulation of NFIC in oxaliplatin- and irinotecan-treated CRC cell lines (40). IKZF2 is a zinc finger transcription factor, which has been shown to act as a tumor suppressor in leukemia (41). Hypermethylated IKZF1 has been reported in CRC tissue, whereas high levels of IKZF2 have been identified in infiltrating lymphocytes in gastric cancer (42, 43). The pathogenic relevance of these regulators to CRC warrants mechanistic evaluation.

Taking all the ELISA validation data from all cohorts interrogated in this study, stool MMP-8, MMP-9, hemoglobin, PGRP-S, haptoglobin, and fibrinogen emerge as the most discriminatory stool proteins for distinguishing CRC from HCs, across cohorts and ethnicities. Of these proteins, stool MMP-8 and MMP-9 exhibited the highest sensitivity (≥0.90) and AUC (0.92, 0.95) in discriminating CRC from HC stool. As a single biomarker, MMP-8 was able to discriminate CRC from HC with 95% accuracy (sensitivity = 90%; specificity = 96%). The diagnostic performance of this single biomarker matches or outperforms FOBT (AUC = 0.77–0.87, specificity = 0.77, and sensitivity = 0.60) as well as FIT (AUC = 0.93, specificity = 0.91, and sensitivity = 0.93 (24, 25, 26), although caution should be exercised as our sample sizes were small, and no head-to-head comparisons were performed.

Elevated serum MMP-8 and stool MMP-9 have indeed been associated with poor prognosis in CRC patients (19, 44). Though MMP-9 was reported in blood, stool, and tumor (45, 46, 47, 48, 49), MMP-8 has not been reported in stool in previous studies. A positive correlation had been found between tumor cell–specific mechanisms such as angiogenesis, epithelial–mesenchymal transition, and increased MMP activity (19, 28). Firebrowse database search suggests high RNA-level FC of 4.83 and 1.95 for MMP-8 and MMP-9, respectively, in CRC (Supplemental Table S4). Compared with our previously published studies (18), the present study adds MMP-8 and PGRP-S as additional stool biomarkers of CRC. More importantly, unlike the previous study, the present study also highlights stool proteins that can reliably distinguish advanced adenomas from HCs.

Specifically, several stool proteins (fibrinogen, MMP-9, hemoglobin, MMP-8, and PGRP-S) significantly discriminate advanced adenomas from HC stool with AUC values from 0.78 to 0.86 (*p* < 0.05). As a single biomarker, stool fibrinogen was able to discriminate adenomas from HC with 86% accuracy (sensitivity = 93%; specificity = 65%). This is significant because few other biomarkers are able to distinguish advanced adenoma from HC with such high diagnostic metrics. FOBT has low sensitivity for both CRC (25–38%) and advanced adenomas (16–31%) (30). Failure to detect adenomas could compromise the utility of FOBT (30). FIT has higher sensitivity for both CRC (61–91%) and advanced adenomas (27–67%) compared with the FOBT but slightly lower specificity (FIT 91–98% *versus* FOBT 98–99%) (30). Additional studies demonstrate that using a second biomarker and FIT improves diagnostic precision (30, 31).

Elevated serum fibrinogen in different digestive tumors may originate from circulating blood as well as tumor epithelial cells (19). Fibrinogen has been reported in blood, stool, and tumor with high RNA FC in CRC in public databases (Supplemental Table S4) (19, 50, 51, 52, 53). Based on the literature reports, fibrinogen could potentially be contributing to tumor growth through focal adhesion kinase activation, which promotes tumor growth by inducing the ubiquitination of p53 in murine CRC (50).

While it is necessary for these findings to be validated in additional patient cohorts, the stool proteins identified in this research demonstrate potential for clinical use in a multitude of ways. Particularly interesting is the observation that stool proteins such as fibrinogen, haptoglobin, hemoglobin, MMP-8, MMP-9, and PGRP-S demonstrate a progressive increase from the HC group to the advanced adenoma group to the CRC group, with significant differences being noted between each successive group. In particular, stool fibrinogen was able to discriminate advanced adenomas from HC with the highest accuracy (AUC = 86%; sensitivity = 93%; and specificity = 65%). Stool fibrinogen may indeed be useful for early screening of high-risk individuals or age-selected population-wide screening for early detection of precancerous lesions. Test positivity would then trigger a colonoscopy. Alternatively, stool proteins with high accuracy and sensitivity values for CRC *versus* HC and CRC *versus* adenoma discrimination (*e.g.*, MMP-8, MMP-9, and PGRP-S, etc.) may be useful for surveillance of subjects with advanced adenomas or high-risk subjects, as well as for monitoring of tumor recurrence following surgery. Moreover, the testing of these stool proteins is readily compatible with point-of-care testing. Thus, home testing or reference laboratory testing of mailed-in stool samples could contribute significantly toward early detection of adenomas/CRC.

This study does have several limitations. Although four CRC cohorts were included in this study, caution should be exercised as the overall cohort sizes were small and all cohorts were cross-sectional in nature. Extended studies in larger cohorts are clearly warranted, particularly in cohorts with well-characterized adenomas, together with disease controls (such as colitis). Validation studies in prospectively collected stool samples and longitudinal studies are also warranted. Furthermore, CRC incidence rates are highest in African Americans; however, this study did not investigate or validate the proposed stool biomarkers in an African-American cohort (54). As such, research is necessary to evaluate the efficacy of these biomarkers in this population, as well as in other high-risk subject categories. Finally, head-to-head comparison studies with current yardsticks such as FOBT and FIT are warranted.

## Data and Material Availability

All data will be freely available to the public, upon request. As provided in the Experimental procedures section, additional data and materials can also be requested upon email.

## Ethics Approval and Consent to Participate

The study was approved by the respective ethics boards of the Mayo Clinic (IRB #16-003882) and the University of Houston (IRB #15192-EX).

## Consent for Publication

Yes, this study consents to publication.

## Supplemental Data

This article contains supplemental data.

## Conflict of Interest

The authors declare no competing interests.
