# Supplementary material for: High Accuracy Stool Biomarkers of Precancerous Colorectal Cancer Identified Using a 2000-Plex Immunoproteomic Screen
Source: Mol Cell Proteomics. 2025 Sep 29;25(3):101079. doi: 10.1016/j.mcpro.2025.101079 (PMC13019079; doi:10.1016/j.mcpro.2025.101079)
Supplement: Supplementary Data [file mmc1.pdf]

Supplementary File Description

Supplementary Figure 1: The consort diagram outlines the overall study design and workflow, beginning with the initial antibody array screen of 2000 proteins in 12 CRC and 12 HC stool samples (Cohort I). Of the 2000 proteins screened, 27 significantly elevated stool proteins were ELISA-validated in Cohort II to confirm initial screening results. 17 of these validated proteins which showed significant discriminatory potential were further ELISA-validated in Cohort III and Cohort IV to ascertain the biomarker potential of the identified stool proteins across cohorts and ethnicities.

Supplementary Figure 2: Association of stool protein biomarker levels with CRC tumor stage (Stage 2, 3 or 4) in Cohort II. *, $p < 0.05$; **, $p<0.01$

Supplementary Table 1: Cohort used for array-based stool screen

Supplementary Table 2: Cohort II used for ELISA validation of stool biomarkers

Supplementary Table 3: ELISA validation of 27 stool proteins significantly elevated in CRC stool, on the L2000 proteomic screen

Supplementary Table 4: Predictive capacity of 27 stool proteins in distinguishing colorectal adenoma from colorectal cancer

Supplementary Table 5. Identification of the most discriminatory stool protein panels using Elastic Net

Supplementary Figure 1

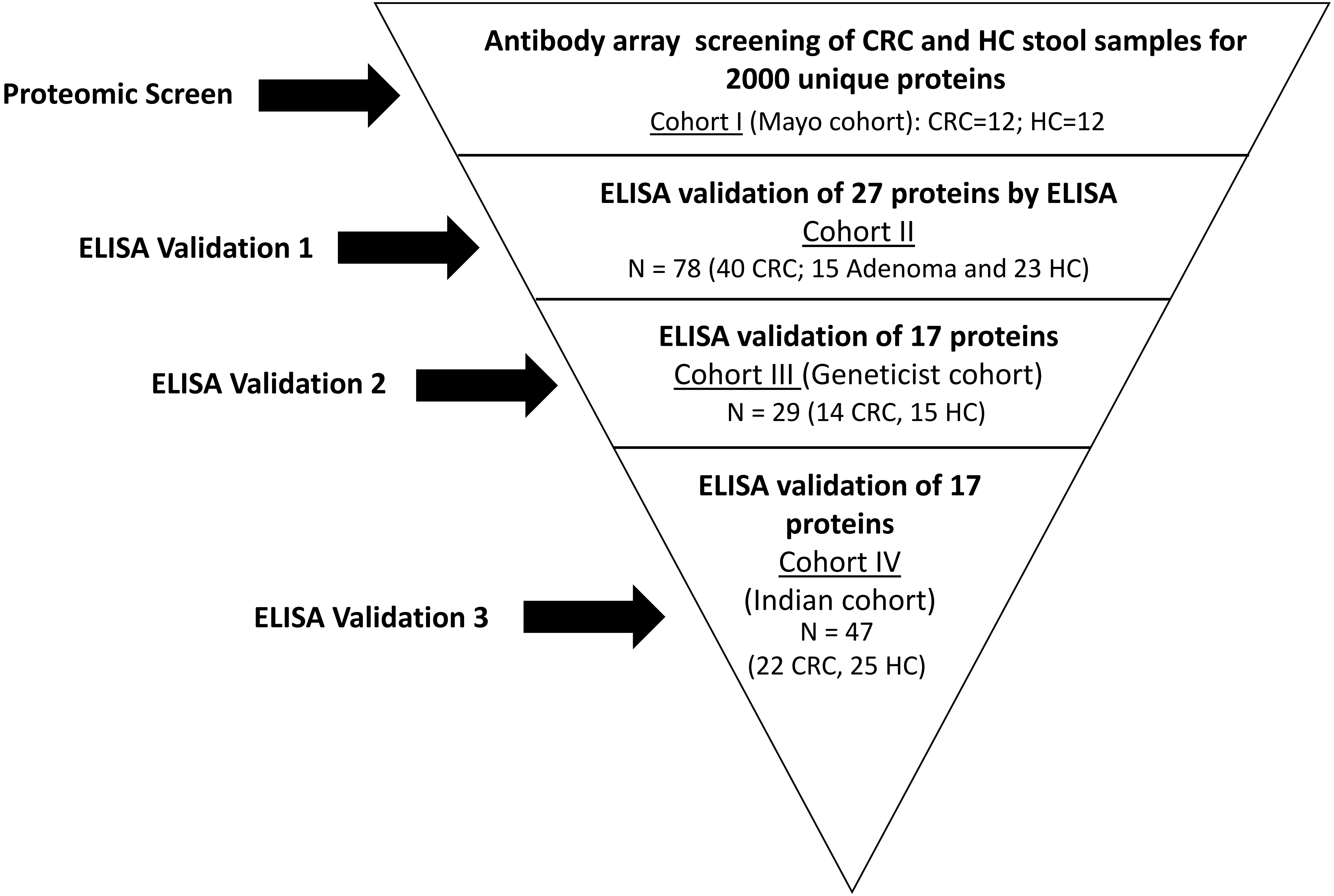

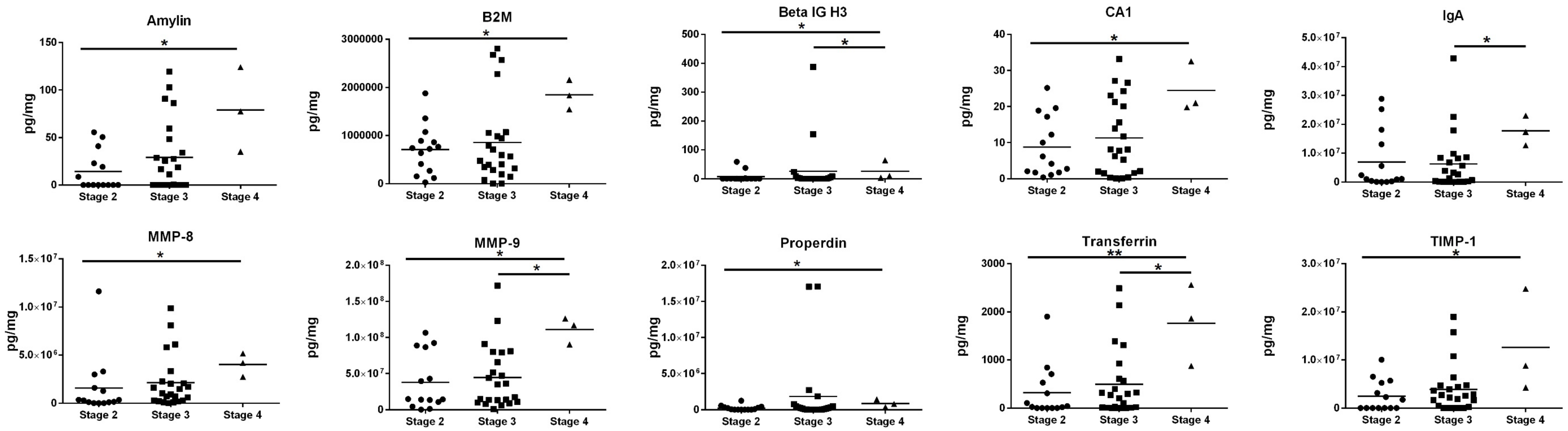

Supplementary Figure 2: Association of stool protein biomarker levels with CRC tumor stage (Stage 2, 3 or 4) in Cohort II. *, $p < 0.05$; **, $p<0.01$

Supplementary Table 1: Cohort used for array-based stool screen

Variable	Category	CRC (N=12)	Healthy Control (N=12)
Age*		67.3±16	69±6.6
Gender; n(%)	Female	4(33.3%)	6(50%)
	Male	8(66.7%)	6(50%)
Grade; n(%)	2	2(16.7%)	N/A
	3	8(66.7%)	N/A
	4	2(16.7%)	N/A
TNM stage; n(%)	1	6(50%)	N/A
	2	2(16.7%)	N/A
	3	2(16.7%)	N/A
	4	2(16.7%)	N/A
Size*		4.7±3.7	N/A
Tumor; n(%)	1	1(8.3%)	N/A
	2	5(41.7%)	N/A
	3	4(33.3%)	N/A
	4	2(16.7%)	N/A
Nodes; n(%)	0	8(66.7%)	N/A
	1	2(16.7%)	N/A
	2	1(8.3%)	N/A
	9	1(8.3%)	N/A
Site; n(%)	Rectum	7(58.3%)	N/A
	Colon	5(41.7%)	N/A
Location; n(%)	Proximal	3(25%)	N/A
	Distal	9(75%)	N/A
Metastasis; n(%)	No	10(83.3%)	N/A
	Yes	2(16.7%)	N/A

**Age and size displayed as mean ± standard deviation*

Supplementary Table 2: Cohort II used for ELISA validation of stool biomarkers

Variable	Category	CRC (N=28)	Adenoma (N=15)	Healthy Control (N=11)
Age*		60.4±12.6	65±13.3	67.5±6.6
Gender; n(%)	Female	9(32.1%)	8(53.3%)	4(36.4%)
	Male	19(67.9%)	7(46.7%)	7(63.6%)
Grade; n(%)	1	N/A	10(66.7%)	N/A
	2	5(17.9%)	5(33.3%)	N/A
	3	21(75%)	N/A	N/A
	4	2(7.1%)	N/A	N/A
TNM stage; n(%)	1	7(25%)	N/A	N/A
	2	5(17.9%)	N/A	N/A
	3	14(50%)	N/A	N/A
	4	2(7.1%)	N/A	N/A
Size*		4.2±3.5	2.8±1.8	N/A
Tumor; n(%)	1	1(3.6%)	N/A	N/A
	2	7(25%)	N/A	N/A
	3	19(67.9%)	N/A	N/A
	4	1(3.6%)	N/A	N/A
Nodes; n(%)	0	12(42.9%)	N/A	N/A
	1	10(35.7%)	N/A	N/A
	2	5(17.9%)	N/A	N/A
	9	1(3.6%)	N/A	N/A
Site; n(%)	Rectum	16(57.1%)	6(40%)	N/A
	Colon	12(42.9%)	9(60%)	N/A
Location; n(%)	Proximal	3(10.7%)	8(53.3%)	N/A
	Distal	25(89.3%)	7(46.7%)	N/A
Metastasis; n(%)	No	26(92.9%)	N/A	N/A
	Yes	2(7.1%)	N/A	N/A

*Age and size displayed as mean ± standard deviation

Note: For adenomas, Grades 1 and 2 refer to low and high grade dysplasia, respectively.

Supplementary Table 3: ELISA validation of 27 stool proteins significantly elevated in CRC stool, on the L2000 proteomic screen

Protein	Company	Catalog number	Dilution	Units	Notes
11b HSD1	MYBIOSOURCE	MBS284409	1:20	pg/mg	Not selected for validation in cross sectional cohort
ABL1	MYBIOSOURCE	MBS944092	1:2	pg/mg	Protein was not detectable by ELISA in preliminary dilution testing
Acrp30 (Adiponectin)	RayBiotech	ELH-Adiponectin-1	1:2	pg/mg	Selected for further validation in cross sectional cohort
Activin C	RayBiotech	ELH-ActivinC-1	1:2	ng/mg	Protein was not detectable by ELISA in preliminary dilution testing
Amylin	MYBIOSOURCE	MBS263609	1:2	ng/mg	Selected for further validation in cross sectional cohort
B2M	RayBiotech	ELH-B2M-1	1:10	pg/mg	Selected for further validation in cross sectional cohort
Beta IG-H3	RayBiotech	ELH-bIGH3-1	1:10	pg/mg	Selected for further validation in cross sectional cohort
C4b	Novus Biologicals	NBP2-70046	1:2	ng/mg	Protein was not detectable by ELISA in preliminary dilution testing
Carbonic anhydrase 1	RayBiotech	ELH-CA1-1	1:200	pg/mg	Selected for further validation in cross sectional cohort
Contactin1	RayBiotech	ELH-CNTN1-1	1:2	ng/mg	Protein was not detectable by ELISA in preliminary dilution testing
D-dimer	RayBiotech	ELH-DDIMER-1	1:20	pg/mg	Not selected for validation in cross sectional cohort
Ferrintin	RayBiotech	ELH-Ferritin-1	1:5	ng/mg	Not selected for validation in cross sectional cohort
Fibrinogen	Immunology Consultants Laboratory	E-80FIB	1:5	ng/mg	Selected for further validation in cross sectional cohort
Haptoglobin	R&D Systems	DHAPG0	1:50	ng/mg	Selected for further validation in cross sectional cohort
Hemoglobin	RayBiotech	ELH-Hgb-1	1:10	ng/mg	Selected for further validation in cross sectional cohort
IgA	Abcam	ab137980	1:5000	ng/mg	Not selected for validation in cross sectional cohort
Integrin α5 (CD49e)	R&D Systems	DY1864-05	1:2	pg/mg	Selected for further validation in cross sectional cohort
Laminin	Abcam	ab119599	1:2	pg/mg	Selected for further validation in cross sectional cohort
Lipocalin 2	RayBiotech	ELH-Lipocalin2-1	1:500	pg/mg	Selected for further validation in cross sectional cohort
Midkine (MDK)	RayBiotech	ELH-MDK-1	1:2	ng/mg	Protein was not detectable by ELISA in preliminary dilution testing
MMP-8	RayBiotech	ELH-MMP8-1	1:10	pg/mg	Selected for further validation in cross sectional cohort
MMP-9	RayBiotech	ELH-MMP9-1	1:100	pg/mg	Selected for further validation in cross sectional cohort
Myeloperoxidase (MPO)	R&D Systems	DY3174	1:4000	pg/mg	Selected for further validation in cross sectional cohort
PGRP-S	R&D Systems	DY2590	1:100	pg/mg	Selected for further validation in cross sectional cohort
Properdin	RayBiotech	ELH-PROPE-1	1:10	pg/mg	Selected for further validation in cross sectional cohort
RBP4	RayBiotech	ELH-RBP4-1	1:100	ng/mg	Selected for further validation in cross sectional cohort
Resistin	R&D Systems	DY1359	1:5	pg/mg	Selected for further validation in cross sectional cohort
S100A12 (EN-RAGE)	R&D Systems	DY1052-05	1:100	pg/mg	Selected for further validation in cross sectional cohort
S100A8/A9	R&D Systems	DY8226-05	1:10000	pg/mg	Selected for further validation in cross sectional cohort
Serpin A4 (Kallistatin)	R&D Systems	DY1669	1:50	pg/mg	Selected for further validation in cross sectional cohort
Serpin A7 (TBG)	R&D Systems	DY8176-05	1:2	pg/mg	Selected for further validation in cross sectional cohort
SOD1	RayBiotech	ELH-SOD1-1	1:500	pg/mg	Selected for further validation in cross sectional cohort
Tenascin	RayBiotech	ELH-TNC-1	1:2	pg/mg	Selected for further validation in cross sectional cohort
TIMP-1	R&D Systems	DY970-05	1:50	pg/mg	Selected for further validation in cross sectional cohort
TIMP-2	R&D Systems	DY971	1:2	pg/mg	Protein was not detectable by ELISA in preliminary dilution testing
Transferrin	RayBiotech	ELH-Trfrn-1	1:50	ng/mg	Selected for further validation in cross sectional cohort
YKL-40 (CHI3L1)	RayBiotech	ELH-CHI3L1-1	1:200	pg/mg	Not selected for validation in cross sectional cohort

Supplementary Table 4: Predictive capacity of 27 stool proteins in distinguishing colorectal adenoma from colorectal cancer

	Comparision of CRC vs Adenoma				
**Protein**	**Cut off**	**AUC**	**Sensitivity**	**Sensitivity^{90}**	**Specificty**
Acrp30	2571952	0.8***	0.83	0.35	0.73
Amylin	9	0.71**	0.55	0.49	0.87
B2M	709073	0.71**	0.53	0.53	0.93
Beta IG-H3	0	0.71****	1.00	0.49	0.43
Carbonic anhydrase 1 (CA:	5	0.76****	0.63	0.55	0.87
Fibrinogen	59602	0.68*	0.58	0.20	0.87
Haptoglobin	42966	0.81****	0.75	0.50	0.80
Hemoglobin	483116	0.77****	0.65	0.50	0.87
IgA	6784125	0.53	0.38	0.20	0.87
Integrin α5 (CD49e)	965	0.67**	0.40	0.42	0.93
Laminin	930849	0.81****	0.83	0.48	0.80
Lipocalin 2 (LCN2)	11569718	0.59 NS	0.48	0.25	0.73
MMP-8	185460	0.87****	0.76	0.65	0.87
MMP-9	9806369	0.84****	0.83	0.52	0.80
Myeloperoxidase (MPO)	333015297	0.88****	0.85	0.69	0.80
PGRP-S	157436	0.85****	0.88	0.64	0.73
Properdin	229659	0.75***	0.50	0.50	0.93
RBP4	16533	0.77***	0.73	0.35	0.87
Resistin	163152	0.74***	0.63	0.42	0.87
S100A12 (EN-RAGE)	461049	0.65****	0.30	0.38	1.00
S100A8,A9	432968831	0.83****	0.70	0.70	0.93
Serpin A7 (TBG)	1	0.64*	0.43	0.25	0.87
Serpin A4 (Kallistatin)	1530451	0.8****	0.65	0.65	0.93
Tenascin C	5386	0.7*	0.58	0.40	0.87
TIMP-1	1602080	0.78****	0.65	0.52	0.87
Transferrin	24	0.77***	0.65	0.58	0.87
YKL-40 (CHI3L1)	5823260	0.74***	0.50	0.50	0.93

Supplementary Table 5. Identification of the most discriminatory stool protein panels using Elastic Net

	5 most discriminatory proteins, in order	AUC Values	Prediction Accuracy	Brier Score	Sensitivity	Specificity
**Panel that discriminates CRC from HC**	MMP8, Hemoglobin, Kallistatin, Fibrinogen, Properdin	0.97 (95% CI: 0.93 - 1)	0.88 (95% CI: 0.77 - 0.95)	0.08	0.83	0.96
**Panel that discriminates HC from Adenoma**	Hemoglobin, Fibrinogen, MMP8, Properdin, PGRPS	0.81 (95% CI: 0.64 - 0.98)	0.72 (95% CI: 0.53 - 0.87)	0.2	0.71	0.73
**Panel that discriminates CRC from Adenoma**	ACP30, Kallistatin, S100A8.A9	0.83 (95% CI: 0.70 - 0.96)	0.74 (95% CI: 0.6 - 0.86)	0.18	0.72	0.82

Shown results were adjusted for age, gender, and ethnicity. LASSO with alpha = 1 (median). Selected biomarkers are listed in orderof importance.

For CRC vs. Adenoma, the demographic predictors (for adjustment) were highly influential in this comparison.

Supplementary Table 6: Protein Levels of 27 stool proteins in CRC, Adenoma and Healthy subjects (Cohort II), as validated by Elisa

	Stool protein, pg/mg, Mean (Median)		Fold change	Comparision of CRC+AA vs HC			
**Protein**	**CRC +AA Mean (median)**	**HC Mean (median)**	**CRC+AA/HC**	**Cut off**	**AUC**	**Sensitivity**	**Specificty**
Acrp30	14273424(7929570)	1260139(398029)	11.3	1846486	0.78	1.00	0.00
Amylin	21(0)	3(0)	7.4	6	0.66	0.45	0.91
B2M	738154(511407)	641816(492200)	1.2	699198	0.51	0.96	0.00
Beta.IG.H3	14(0)	0(0)	84.0	1	0.6	0.21	0.95
CA1	9(5)	2(1)	3.9	4	0.74	0.65	0.60
CD49E	38810(0)	0(0)	0.0	482	0.65	0.30	1.00
CHI3L1	10530027(2185634)	1060034(186416)	9.9	644399	0.82	1.00	0.00
ENRAGE	397033(0)	0(0)	0.0	230525	0.6	0.21	1.00
Fibrinogen	78711(45307)	7211(3049)	10.9	15515	0.87	0.98	0.00
Haptoglobin	657223(83846)	4100(1321)	160.3	34216	0.85	0.98	0.04
Hemoglobin	584169(483116)	42062(1735)	13.9	19383	0.88	0.92	0.30
IgA	6625534(2365083)	1998991(264160)	3.3	2518559	0.68	0.90	0.34
Kallistatin	4286632(1522922)	72951(0)	58.8	1	0.78	0.60	0.91
Laminin	1802581(1163199)	901611(838922)	2.0	1489734	0.68	1.00	0.00
LCN2	15606902(8601014)	4901538(3535274)	3.2	6959336	0.78	0.98	0.00
MMP8	1547407(295986)	10524(0)	147.0	44644	0.9	0.83	0.78
MMP9	36171622(14193106)	2500668(1543843)	14.5	2404805	0.88	0.98	0.00
MPO	1739999651(671998972)	483089795(212444218)	3.6	303500000	0.73	0.94	0.13
PGRPS	1614813(451436)	84292(30498)	19.2	67567	0.88	1.00	0.21
Properdin	894675(69428)	0(0)	0.0	1	0.81	0.61	1.00
RBP4	43200(19224)	4634(2284)	9.3	5708	0.84	1.00	0.04
Resistin	300191(152655)	117677(93149)	2.6	161968	0.66	0.98	0.00
S100A8,A9	839670336(437157100)	169688206(0)	4.9	531000000	0.77	0.85	0.52
Serpin A7	28610(0)	0(0)	0.0	1	0.67	0.32	1.00
Tenascin C	39031(4249)	2169(1384)	18.0	4646	0.71	0.96	0.00
TIMP1	3053503(1602080)	147359(0)	20.7	61447	0.76	0.58	0.95
Transferrin	432(24)	16(0)	26.3	4	0.8	0.65	0.95

1: The validation cohort of 78 subjects was comprised of 40 CRC patients, 15 adneoma patients, and 23 healthy controls.

Supplementary Table 7: Protein Levels of 17 stool proteins in CRC, Adenoma and Healthy subjects, as validated by Elisa (in 3 cohorts, combined)

	Stool protein, pg/mg, Mean (Median)		Fold change		Comparision of CRC vs HC		
**Protein**	**CRC +AA Mean (median)**	**HC Mean (median)**	**CRC+AA/HC**	**Cut Off**	**AUC**	**Sensitivity**	**Specificty**
Acrp30	8626850(486255)	460057(15)	18.75	69	0.75	0.93	0.2
Amylin	16(4)	4.26(2.9)	3.77	0.2294	0.58	0.59	0.36
CHI3L1	6364527(213634)	3870099(26)	1.644	34	0.78	0.98	0.14
CNTN1	0.15(0)	0.09(0)	1.58	0.004938	0.48	0.01	0.98
Fibrinogen	47934(6246)	2649(45)	18.09	144	0.79	0.96	0.14
Haptoglobin	397546(2271)	1504(0)	264.18	116	0.82	0.901	0.5
Hemoglobin	2585748(553117)	149149(0)	17.33	162894	0.86	0.87	0.58
Kallistatin	2590892(189)	26632(0)	97.28	0.2254	0.74	0.51	0.96
MMP-8	935268(8189)	3843(0)	243.35	4	0.87	0.79	0.79
MMP-9	21862758(2507261)	912974(21)	23.94	2083700	0.79	0.94	0.44
MPO	1051663741(193086054)	176367491(2519)	5.96	8835	0.76	0.96	0.28
PGRPS	975999(88379)	30774(1.9)	31.71	3	0.81	0.93	0.53
Properdin	540761.63(7.9)	0.2820(0)	1917085.37	0.2252	0.81	0.56	0.95
RBP4	27200(8256)	1800(246)	15.1	157799	0.79	1	0.01587
S100A8/A9 pg/mg	507511425(97557055)	61951112(0)	8.19	8283	0.79	0.83	0.65
TIMP-1	1845555(24.9)	53798(0)	34.3	12	0.75	0.53	0.95
Transferrin	1059.09(19)	6.47(0)	163.64	2	0.78	0.6	0.96
